# P-932. Re-classification of Endocarditis Diagnoses in Patients who Inject Drugs using the 2023 Duke-ISCVD Microbiologic Criteria

**DOI:** 10.1093/ofid/ofae631.1123

**Published:** 2025-01-29

**Authors:** David M Henson, Talal Alnabelsi, Bennett Collis, Evan Hall, Bobbi Jo Stoner, Sami El-Dalati

**Affiliations:** University of Kentucky, Lexington, Kentucky; University of Kentucky, Lexington, Kentucky; University of Kentucky, Lexington, Kentucky; University of Kentucky, Lexington, Kentucky; University of Kentucky, Lexington, Kentucky; University of Kentucky, Lexington, Kentucky

## Abstract

**Background:**

In 2023, the Duke Criteria for infectious endocarditis (IE) were modified for a second time. Many criteria were adjusted with significant changes to the microbiologic criteria, introduction of new imaging criteria, and greater clarity of predisposing conditions contributing to the IE diagnosis. The changes to the microbiological criteria included expanding the list of typical organisms and adding cultures from a sterile site as a possible minor criterion. Other studies have assessed the impact of the updated criteria on the diagnosis of IE in large patient subsets. However, none have evaluated the effect of the criteria on diagnosis specifically in patients who inject drugs.

**Figure:**
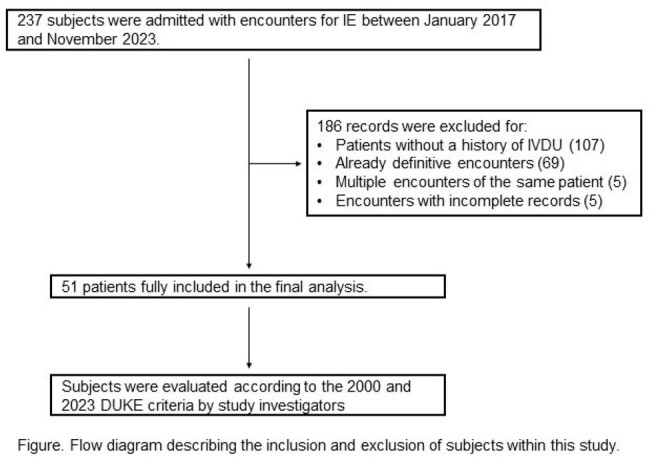

**Methods:**

Patients were identified through pre-existing data from a previous study and from the records of the institutional multidisciplinary endocarditis team. Charts of 237 patients with a preliminary classification as possible or rejected IE were reviewed by study investigators in the electronic medical record based on the 2000 modified Duke Criteria, the 2023 Duke-ISCVD Criteria, and for a history of intravenous drug use (IVDU).

**Figure:**
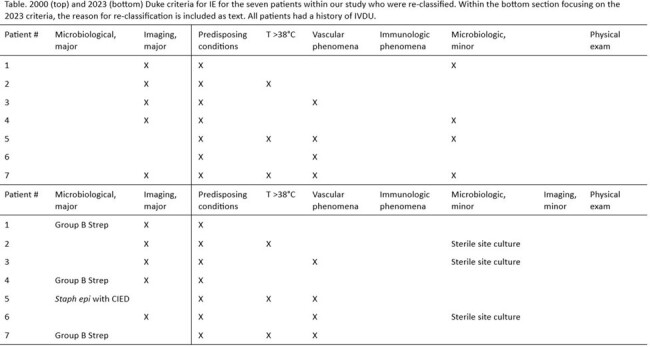

**Results:**

Between January 1st, 2017 and November 1st, 2023, 51 patients with possible or rejected IE by the modified Duke Criteria and a history of IVDU were identified. Seven (13.7%) were re-classified from possible IE to definite IE based on the 2023 Duke-ISCVD criteria. All upgraded diagnoses were secondary to the new microbiologic criteria. Three patients were re-classified due to Streptococcus agalactiae bacteremia. An additional patient was re-classified due to Staphylococcus epidermidis bacteremia in the context of intra-cardiac material. The remaining three patients were re-classified due to positive cultures from sterile sites other than blood. All three patients received antibiotics prior to blood culture collection.

**Conclusion:**

Compared to the modified Duke Criteria, applying the 2023 Duke-ISCVD criteria for IE resulted in the re-classification in 13.7% of patients with a history of IVDU from possible or rejected IE to definite IE. All upgraded diagnoses were secondary to changes in the microbiologic criteria. The 2023 Duke-ISCVD criteria may lead to increased diagnosis of definite IE in patients who inject drugs.

**Disclosures:**

**All Authors**: No reported disclosures

